# CaMYB80 enhances the cold tolerance of pepper by directly targeting *CaPOA1*

**DOI:** 10.1093/hr/uhae219

**Published:** 2024-08-06

**Authors:** Jiachang Xiao, Dong Wang, Le Liang, Minghui Xie, Yi Tang, Yun-Song Lai, Bo Sun, Zhi Huang, Yangxia Zheng, Huanxiu Li

**Affiliations:** College of Horticulture, Sichuan Agricultural University, Chengdu 611130, China; College of Horticulture, Sichuan Agricultural University, Chengdu 611130, China; College of Horticulture, Sichuan Agricultural University, Chengdu 611130, China; College of Horticulture, Sichuan Agricultural University, Chengdu 611130, China; College of Horticulture, Sichuan Agricultural University, Chengdu 611130, China; College of Horticulture, Sichuan Agricultural University, Chengdu 611130, China; College of Horticulture, Sichuan Agricultural University, Chengdu 611130, China; College of Horticulture, Sichuan Agricultural University, Chengdu 611130, China; College of Horticulture, Sichuan Agricultural University, Chengdu 611130, China; College of Horticulture, Sichuan Agricultural University, Chengdu 611130, China

## Abstract

Cold temperatures negatively impact crop yield and quality, posing significant limitations to the advancement of the vegetable industry. MYB transcription factors are pivotal in enhancing plant resilience against various abiotic stresses, including low-temperature stress. Pepper (*Capsicum annuum* L.) is a nutrient-rich vegetable crop sensitive to low temperatures. This study aimed to determine the function of *CaMYB80* in the cold stress response of pepper through virus-induced silencing. The study also conducted heterologous expression of *CaMYB80* in Arabidopsis and tomato plants. The results showed that *CaMYB80* could respond to low-temperature stress in pepper. *CaMYB80* was localized in the nucleus and cytoplasm and exhibited transcriptional activation ability. Moreover, *CaMYB80* silencing decreased cold tolerance in pepper, while its heterologous overexpression increased cold tolerance in Arabidopsis and tomato. Further analysis showed that CaMYB80 interacted with *CaPOA1* (peroxidase N1-like). Similarly, the expression of *CaPOA1* also responded to low-temperature stress. Overexpression of *CaPOA1* enhanced freezing tolerance in Arabidopsis, while its silencing reduced cold stress tolerance in pepper. Furthermore, overexpression of *CaMYB80* in Arabidopsis and tomato could increase the activity of peroxidases and the expression levels of genes in the ICE-CBF-COR (inducer of CBF expression, C-repeat binding factor, cold-responsive) regulatory network. In conclusion, our research results indicate that CaMYB80 enhances pepper cold tolerance by interacting with *CaPOA1* to increase peroxidase activity and influence the expression of ICE-CBF-COR related genes.

## Introduction

Low temperature stress is a prevalent abiotic factor that impacts plant growth and distribution, leading to diminished crop yield and quality [1]. Cold stress can be classified as either chilling (0–15°C) stress or freezing (<0°C) stress. Chilling stress inhibits plant growth and development, while freezing stress disrupts cell structure, leading to cell death [2]. Plants have developed sophisticated regulatory mechanisms, encompassing numerous signaling pathways, to promptly detect and efficiently counteract cold stress [3]. These pathways constitute an intricate network involving a multitude of genes encoding both structural and regulatory proteins, which directly or indirectly shield plants from the adverse effects of low temperatures [4, 5].

Transcription factors (TFs) play a crucial role as regulatory proteins, governing the expression of target genes by binding to specific *cis*-regulatory elements within their promoter regions [6]. Under cold stress conditions, a variety of TFs are activated, such as CBF, bHLH, MYB, and NAC families. These TFs stimulate the transcription of downstream cold-responsive (COR) genes, thereby propagating and intensifying the cold signal and initiating a cascade of physiological responses [6–8]. As one of the most extensive families of TFs, MYB TFs exert significant regulatory control over plant responses to abiotic stress by binding to MYB elements and modulating the expression of relevant genes [1]. Named after their highly conserved DNA-binding domain located at the N-terminal region, termed the MYB domain, these TFs typically contain one to four repeats of the R motif [9]. The MYB TF family can be categorized into four subfamilies based on the number of R repeats present in their sequence: 1R-MYB (R1/R2, R3-MYB), 2R-MYB (R2R3-MYB), 3R-MYB (R1R2R3-MYB), and 4R-MYB (R1/R2-MYB) [8–10].

Within these subfamilies, R2R3-MYB has garnered considerable attention owing to its multifaceted roles in plant biology. Numerous studies have elucidated the involvement of certain R2R3-MYB TFs, such as *At*MYB15, *At*MYB88, *At*MYB44, *Gm*MYB92, *Ta*MYB2A, and *Os*MYB2, in modulating plant responses to various environmental stresses, including drought, salinity, and cold [11–16]. Additionally, research has demonstrated that *Dg*MYB2 can enhance chrysanthemum’s cold tolerance by regulating *DgGPX1* [1]. Moreover, overexpression of *SlMYB102* in tomato has been shown to upregulate *SlP5CS* and *SlAPX2* genes under low temperatures, thereby bolstering tomato plants’ cold tolerance via the CBF and proline synthesis pathways [17].

During episodes of low-temperature stress, the equilibrium of reactive oxygen species (ROS) within plants becomes disturbed, resulting in swift ROS buildup and subsequent oxidative harm [18, 19]. Peroxidases (POD, PRX, and POA) play pivotal roles as antioxidant enzymes within the ROS detoxification system. These enzymes facilitate the oxidation of various substrates using H_2_O_2_ as an electron donor, thereby curtailing ROS accumulation induced by abiotic stress and preserving membrane integrity [20, 21]. Nevertheless, the precise regulatory pathways governing the activity of antioxidant enzymes mediated by MYB transcription factors in pepper remain largely unexplored.

Pepper (*Capsicum annuum* L.), an important vegetable crop, is an annual or perennial herbaceous plant belonging to the Solanaceae family. It thrives under normal growth temperatures of 20–30°C and is susceptible to various environmental stresses [6]. Notably, unseasonably cold conditions during winter and spring can significantly impede the growth and maturation of pepper plants, leading to reduced productivity and economic value [22]. Therefore, investigating the response of pepper to low-temperature stress is imperative for enhancing both its quality and yield. In this study, we isolated a low-temperature-induced R2R3-MYB TF, *CaMYB80*, from pepper and investigated its role in regulating low-temperature tolerance through virus-induced gene silencing (VIGS). The analysis was also conducted in Arabidopsis and tomato plants. We found that the C-terminal region of *CaMYB80* possesses transcription activation ability, allowing it to directly bind the *CaPOA1* promoter and activate its expression to enhance plant cold tolerance. Additionally, the heterologous overexpression of *CaPOA1* also improved the cold resistance of Arabidopsis. In summary, our findings prove that *CaMYB80* plays a crucial role in plant cold stress by directly regulating *CaPOA1* expression, thereby enhancing peroxidase activity.

## Results

### *CaMYB80* is responsive to low-temperature stress

Pepper leaves exposed to cold conditions (0 h, 6 h, and 24 h treatment at 4°C) underwent transcriptome analysis, with data accessible under the NCBI accession number PRJNA778231. The findings revealed a notable upregulation of 30 MYB gene family members at the transcriptional level following low-temperature treatment (Fig. S1a, see online supplementary material), with statistical significance (*P* < 0.05). Among these genes, LOC107845708 (GenBank accession number: NC_061120) demonstrated substantial induction (log2[fold change] = 1.62 ~ 1.73) and was consequently earmarked for further investigation.

**Figure 1:**
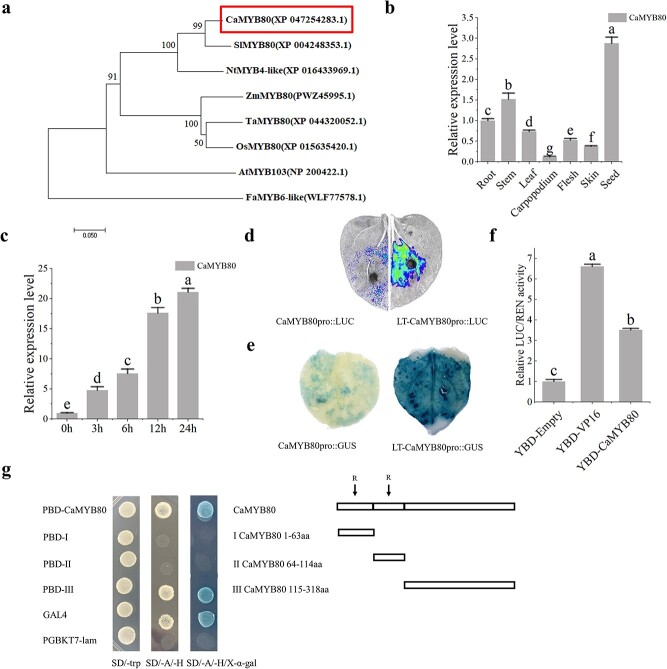
*CaMYB80* is responsive to low-temperature stress. Values are means ± SD from three independent experiments. Values with different letters above the bars are significantly different at *P* < 0.05. **(a)** Phylogenetic tree analysis of the CaMYB80 family from pepper (*Capsicum annuum*), tomato (*Solanum lycopersicum*), tobacco (*Nicotiana tabacum*), corn (*Zea mays*), rice (*Oryza sativa*), arabidopsis (*Arabidopsis thaliana*), wheat (*Triticum aestivum*), and strawberry (*Fragaria ananassa*). **(b)** Relative expression levels of *CaMYB80* in the roots, stems, leaves, carpopodiums, flesh, skins and seeds of wild-type (WT) pepper, as determined by RT–qPCR. **(c)** Relative expression levels of *CaMYB80* in pepper under cold conditions (4°C). **(d)** Luciferase complementation imaging (LCI) of the *CaMYB80* native promoter after transient expression in tobacco; the results of the control (25°C/22°C day/night) and cold (4°C for 6 h) treatment groups are compared of the *CaMYB80*. **(e)** GUS staining of the *CaMYB80* native promoter after transient expression in tobacco. **(f)** Transcriptional transactivation in tobacco. **(g)** Transcriptional transactivation in yeast; the right panel shows the *CaMYB80* fragment used for testing.

The full-length cDNA sequence of *CaMYB80* comprised a 957 bp open reading frame (ORF) encoding 318 amino acids, with a calculated molecular weight of 35.16 kDa and a theoretical isoelectric point of 8.32. Analysis of the amino acid sequence revealed the presence of two SANT/MYB conserved domains (13~63, 66~114), indicating that CaMYB80 conforms to the typical structure of an R2R3-MYB transcription factor. Phylogenetic assessment demonstrated a high degree of sequence similarity between CaMYB80 and the SlMYB80 sequence from *Solanum lycopersicum* (Fig. 1a).

Expression analysis of *CaMYB80* across various tissues revealed a notably high expression level in seeds, followed by the stem, while the lowest expression was observed in Carpopodiums (Fig. 1b). We also found that the expression of *CaMYB80* increased continuously with the low-temperature treatment duration, reaching its highest level at 24 h (Fig. 1c). To further investigate this, we inserted the natural promoter of *CaMYB80* (2000 bp) into a vector containing LUC and GUS reporters for analysis. Plant luciferase *in vivo* imaging and GUS staining results revealed that the low-temperature treatment boosted the activity of the *CaMYB80* promoter (Fig. 1d and e). This was further confirmed by quantifying LUC and GUS enzyme activities (Fig. S1b and c).

To validate the transcriptional activity of CaMYB80, we employed a dual-luciferase reporter gene system. Our findings demonstrated that the experimental group harboring the pBD-CaMYB80 effector vector exhibited notably higher LUC/REN values compared to the negative control (pBD-empty), suggesting that CaMYB80 possesses transcriptional activation capability (Fig. 1f). To determine the transcriptional activation region of CaMYB80, we conducted yeast two-hybrid assay using the recombinant plasmids containing the corresponding fragments. It was found that the yeast cells transformed with the complete ORF sequence of CaMYB80 (1–318 aa) and the C-terminal sequence of CaMYB80 (115–318 aa) grew normally on SD/−Ade/-His medium (Fig. 1g), indicating that the C-terminus of CaMYB80 has transcriptional activation ability.

### *CaMYB80* silencing reduces the cold tolerance of pepper

Firstly, we explored the function of *CaMYB80* through VIGS. The results showed that unlike the TRV2:00 and TRV2:CaMYB80 plants, which had green leaves, leaf whitening was observed in TRV2: CaPDS plants after 3 weeks of transient transformation, demonstrating the reliability of the experiment (Fig. S2a, see online supplementary material). Subsequently, we measured the silencing efficiency using RT-qPCR and found that the silencing efficiency was close to 70% (Fig. S2b, see online supplementary material).

**Figure 2:**
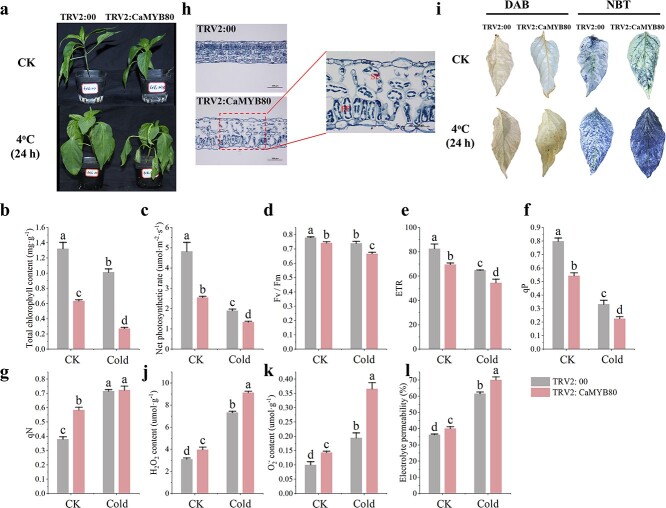
*CaMYB80* silencing reduces the cold tolerance of pepper. Values are means ± SD from three independent experiments. Values with different letters above the bars are significantly different at *P* < 0.05. **(a)** Comparison phenotypes between CaMYB80-silenced plants and control plants. **(b)** Total chlorophyll content. **(c)** Net photosynthetic rate. **(d)** Fv/Fm. **(e)** ETR. **(f)** qP. **(g)** qN. **(h)** Transverse sections of leaves of CaMYB80-silenced plants and control plants at low temperature. PP: palisade tissue; SP: spongy tissue. **(i)** NBT and DAB histochemical staining. **(j)** H_2_O_2_ content. **(k)** O_2_^−^ content. **(l)** Electrolyte permeability.

Under normal temperature conditions, TRV2:CaMYB80 plants exhibited reduced growth compared to TRV2:00 plants (Fig. 2a), as evidenced by significant reductions in their total chlorophyll content, net photosynthetic rate (Pn), potential photochemical efficiency (Fv/Fm), electron transport rate (ETR), and photochemical quenching (qP) (Fig. 2b–f). After a 24-hour treatment at 4°C, TRV2:CaMYB80 plants suffered more severe photosynthetic damage compared to TRV2:00 plants, with significant decreases in their chlorophyll content, Pn, Fv/Fm, ETR, and qP. However, non-photochemical quenching (qN) showed no significant difference between the two groups (Fig. 2b–g).

Because the TRV2:CaMYB80 plants exhibited lower photosynthetic capacity, we studied their leaf anatomy. As shown in Fig. 2h, after 3 days of low-temperature treatment, TRV2:CaMYB80 leaves exhibited a more loosely arranged cell structure with disordered arrangement of fence tissues and larger and less tightly arranged intercellular spaces in sponge tissues compared to TRV2:00 plants. This indicated that TRV2:CaMYB80 plants suffered more severe leaf damage under low-temperature stress.

We utilized staining techniques with 3,3′-diaminobenzidine (DAB) and nitroblue tetrazolium (NBT) to assess the accumulation of H_2_O_2_ and O_2_^−^ in leaves, respectively. Compared to TRV2:00 plants, the leaves of TRV2:CaMYB80 plants exhibited a larger staining area under low-temperature stress (Fig. 2i), indicating a significant increase in the H_2_O_2_ and O_2_^−^ levels (Fig. 2i–g). Additionally, there was a significant increase in electrolyte permeability of TRV2:CaMYB80 plants (Fig. 2k), suggesting increased cell membrane damage in TRV2:CaMYB80 plants under low-temperature stress.

Due to variations in the aforementioned physiological indicators induced by low-temperature stress, we conducted measurements of superoxide dismutase (SOD), peroxidase (POD), catalase (CAT), proline (Pro), soluble protein (Sp), and malondialdehyde (MDA) levels in pepper seedlings. Following a 24-hour treatment at 4°C, the SOD, POD, CAT, and Pro levels exhibited significant decreases, while the MDA level showed a significant increase in TRV2:CaMYB80 plants compared to control plants. However, there was no significant difference in the Sp level between the two groups (Fig. S2c–h, see online supplementary material).

Overall, our results demonstrate that *CaMYB80* silencing reduces the ability of pepper plants to cope with ROS overproduction, resulting in increased oxidative damage.

### Ectopic expression of *CaMYB80* enhances Arabidopsis cold tolerance

Due to the recalcitrance of pepper in tissue culture, this experiment chose two model plants, Arabidopsis and tomato, for gene functional validation. To delve deeper into the role of *CaMYB80*, we first obtained T3 generation stable transgenic Arabidopsis plants. From these, we identified three transgenic Arabidopsis lines, designated OE3, OE9, and OE10, for further investigation. Relative to WT plants, these selected transgenic lines exhibited elevated expression levels of *CaMYB80* (Fig. S3a, see online supplementary material). Subsequent non-acclimated (NA, −7°C) and cold-acclimated (CA, −10°C, plants exhibit stronger cold tolerance after cold acclimation, hence we chose to conduct tests at −10°C) conditions revealed that the heterologous overexpression of *CaMYB80* significantly enhanced the freezing tolerance of Arabidopsis, leading to increased survival rates of transgenic Arabidopsis under both NA and CA conditions (Fig. S3c, see online supplementary material).

**Figure 3:**
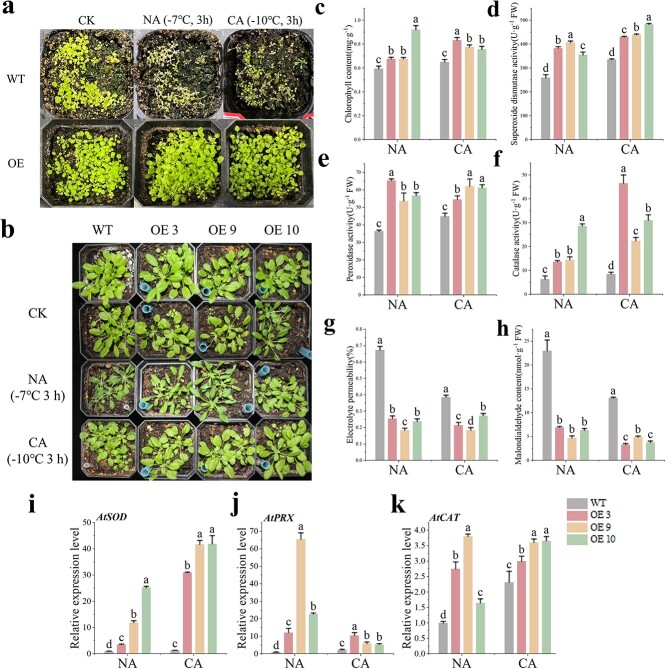
Ectopic expression of *CaMYB80* enhances Arabidopsis cold tolerance. Values are means ± SD from three independent experiments. Values with different letters above the bars are significantly different at *P* < 0.05. **(a)** Survival rate phenotypes of WT Arabidopsis and transgenic lines (OE3, OE9, O10). **(b)** Comparison phenotypes between WT Arabidopsis and transgenic lines (OE3, OE9, OE10). **(c)** Total chlorophyll content. **(d)** Superoxide dismutase activity. **(e)** Peroxidase activity. **(f)** Catalase activity. **(g)** Electrolyte permeability. **(h)** Malondialdehyde content. **(i)–(k)** Expression levels of *AtSOD*, *AtPRX*, and *AtCAT* in WT Arabidopsis and transgenic lines (OE3, OE9, OE10).

Compared to WT plants, the overexpression of *CaMYB80* notably improved the freezing tolerance of Arabidopsis under both NA and CA conditions (Fig. 3b). Additionally, *CaMYB80* overexpression increased the total chlorophyll content, as well as the SOD, POD, and CAT activities of Arabidopsis plants (Fig. 3c–f). Electrolyte permeability and MDA analysis results further indicated that OE-CaMYB80 Arabidopsis had less membrane damage than WT plants under freezing stress (Fig. 3g and h). After cold acclimation, the WT plants showed reduced membrane oxidative damage caused by freezing stress. We also verified the expression level of *AtSOD*, *AtPRX*, and *AtCAT* genes in Arabidopsis under NA and CA treatments. The findings indicated a significant elevation in the expression levels of *AtSOD*, *AtPRX*, and *AtCAT* genes in Arabidopsis under both NA and CA treatments upon overexpression of *CaMYB80*, aligning with the observed physiological indicators (Fig. 3i–k).

These findings indicate that *CaMYB80* has the capacity to bolster the freezing tolerance of Arabidopsis by amplifying the activity of antioxidant enzymes and the expression of associated genes, consequently mitigating ROS accumulation and membrane damage induced by freezing stress. Consequently, *CaMYB80* assumes a positive regulatory role in Arabidopsis’ response to freezing stress.

### Ectopic expression of *CaMYB80* enhances the cold tolerance of tomato

We further overexpressed *CaMYB80* in tomato to obtain T1 transgenic tomato plants. We selected three transgenic tomato lines (OE1, OE4, and OE9) with high expression of *CaMYB80* via PCR and RT-qPCR for further low-temperature experiments (Fig. S4a and b, see online supplementary material).

**Figure 4:**
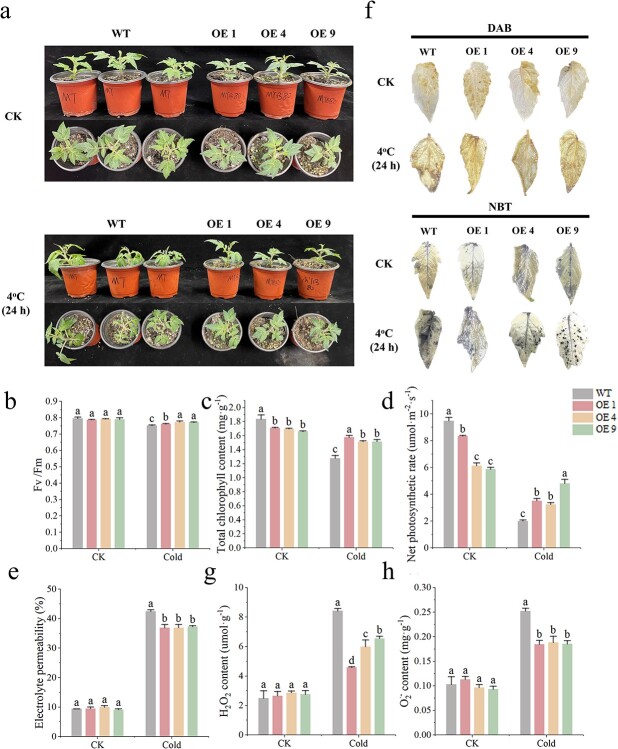
Ectopic expression of *CaMYB80* enhances the cold tolerance of tomato. **(a)** Comparison of phenotypes between WT tomatoes and transgenic tomatoes (OE1, OE4, OE9). **(b)** Fv/Fm. **(c)** Total chlorophyll content. **(d)** Net photosynthetic rate. **(e)** Electrolyte permeability. **(f)** NBT and DAB histochemical staining. **(g)** H_2_O_2_ content. **(h)** O_2_^−^ content. Values are means ± SD from three independent experiments. Values with different letters above the bars are significantly different at *P* < 0.05.

Under cold stress conditions, both WT tomato plants and three lines of tomato overexpressing *CaMYB80* (OE1, OE4, and OE9) exhibited leaf wilting, albeit with more pronounced wilting observed in the WT plants (Fig. 4a). The overexpression of *CaMYB80* conferred enhanced tolerance to low-temperature stress in tomato plants, mirroring the findings observed in transgenic Arabidopsis. Assessment of potential photosynthetic efficiency of PSII (Fv/Fm) revealed that, under low temperatures, CaMYB80-overexpressing tomato plants exhibited lesser inhibition of Fv/Fm (Fig. 4b), along with higher chlorophyll content and Pn levels (Fig. 4c and d) in their leaves compared to WT plants. Under normal temperature conditions, there was no notable variance in electrolyte permeability between the CaMYB80-overexpressing and WT plants. However, under low-temperature conditions, the electrolyte permeability of the WT tomato plants was significantly higher than that in the CaMYB80-overexpressing tomato plants (Fig. 4e). The changes in H_2_O_2_ and O_2_^−^ contents also followed a similar pattern, with more staining observed in the leaves of the WT plants stained with DAB and NBT under low temperature compared to the CaMYB80-overexpressing plants (Fig. 4f). This was further confirmed by the quantitative analysis of H_2_O_2_ and O_2_^−^ contents (Fig. 4g and h). Furthermore, the activity of ROS scavenging enzymes, as well as the levels of Pro and MDA, were measured. Compared to the WT plants, the CaMYB80-overexpressing tomato plants showed significantly increased SOD, POD, and CAT activities, enhanced proline content, and reduced accumulation of MDA under low-temperature stress (Fig. S5a–e, see online supplementary material).

### *CaPOA1* is responsive to low-temperature stress

**Figure 5:**
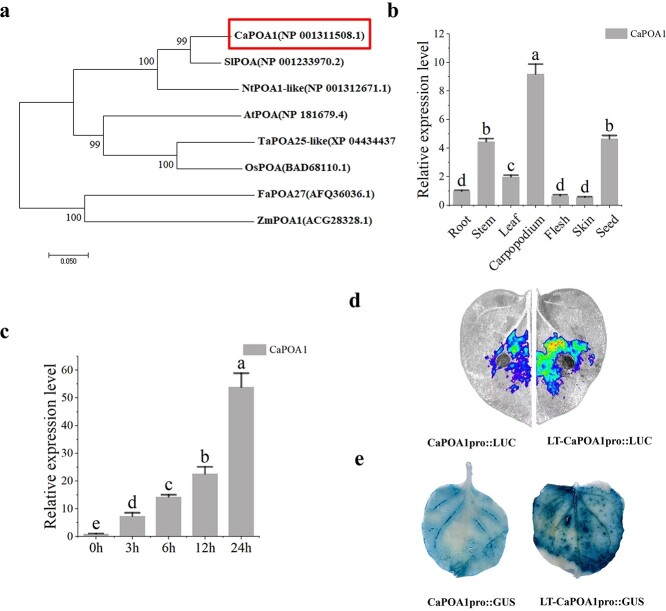
*CaPOA1* is responsive to low-temperature stress. Values are means ± SD from three independent experiments. Values with different letters above the bars are significantly different at *P* < 0.05. **(a)** Phylogenetic tree analysis of the CaPOA1 family from (*Capsicum annuum*), tomato (*Solanum lycopersicum*), tobacco (*Nicotiana tabacum*), corn (*Zea mays*), rice (*Oryza sativa*), arabidopsis (*Arabidopsis thaliana*), wheat (*Triticum aestivum*), and strawberry (*Fragaria ananassa*). **(b)** Relative expression levels of *CaPOA1* in the roots, stems, leaves, carpopodiums, flesh, skins and seeds of WT pepper, as determined by RT–qPCR. **(c)** Relative expression levels of *CaPOA1* in WT pepper under cold conditions (4°C). **(d)** Luciferase complementation imaging (LCI) of the *CaPOA1* native promoter after transient expression in tobacco. **(e)** GUS staining of the *CaPOA1* native promoter after transient expression in tobacco. The results of the control (25°C/22°C day/night) and cold (4°C for 6 h) treatment groups are compared of the *CaPOA1*.

The complete cDNA sequence of *CaPOA1* (peroxidase N1-like, LOC107840027) contained an ORF spanning 996 base pairs, which encoded a protein of 332 amino acids. The protein exhibited a molecular weight of 36.07 kDa and a theoretical isoelectric point of 6.88. Phylogenetic analysis indicated a close relationship between CaPOA1 and SlPOA (*S. lycopersicum*) (Fig. 5a).

Analysis of *CaPOA1* expression across various tissues revealed its highest expression in the Carpopodium, followed by the stem, while the lowest expression was observed in the skin (Fig. 5b). Additionally, we noted a steady increase in *CaPOA1* expression with prolonged low-temperature treatment, peaking at 24 hours (Fig. 5c). To investigate further, we inserted the native promoter of *CaPOA1* (2000 bp) into a vector containing LUC and GUS reporter for detection. Through *in vivo* imaging and GUS staining, we found that low-temperature stress enhanced the activity of the *CaPOA1* promoter (Fig. 5d and e). We validated these results through LUC reporter gene assays and GUS enzyme activity tests (Fig. S6a and b, see online supplementary material).

### CaMYB80 binds to the promoter of *CaPOA1*

The reduction of ROS is associated with various antioxidant enzymes. We further analyzed all *cis*-element analysis in the promoter regions of *CaSOD*, *CaPOA1*, *CaCAT*, *CaAPX1*, and *CaDHAR2*. Based on their functional associations, we categorized them into four categories (Fig. S7, see online supplementary material): biotic and abiotic stress-related elements (LTR, TC-rich repeats, and MBS), hormone-responsive elements (ABRE, TGACG-motif, P-box, TGA-element, CGTCA-motif, and TCA-element), light-responsive elements (MRE, G-box, GT1-motif, GA-motif, ACE, AE-Box, GATA-motif, I-box, and TCCC-motif), and MYB-response elements (MYB). Our results indicate that the promoters of the *CaSOD*, *CaPOA1*, and *CaCAT* contain MYB elements, but only the natural promoter of *CaPOA1* (186–200 bp) includes a binding site for CaMYB80 (AATAATTAGGTAACC). Therefore, we speculate that CaMYB80 can directly regulate the expression of *CaPOA1*.

**Figure 6:**
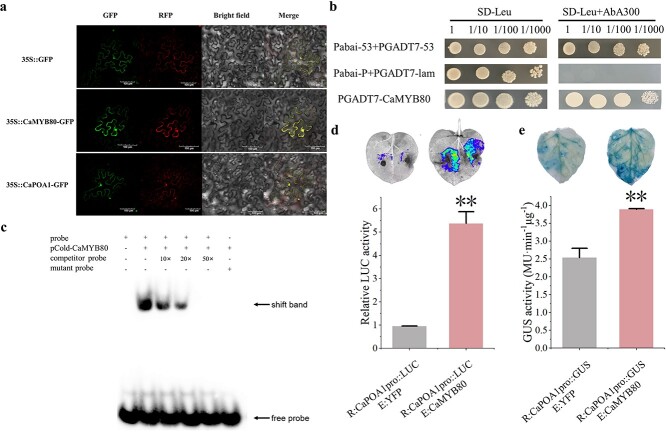
CaMYB80 binds to the promoter of *CaPOA1*. **(a)** Subcellular localization; scale bars = 10 μm. **(b)** Results of the Y1H experiment. **(c)** EMSA, from left to right: 6-FAM labeled prope; CaMYB80 and 6-FAM labeled probe; CaMYB80, 6-FAM labeled probe and 10× unlabeled probe; CaMYB80, 6-FAM labeled probe and 20× unlabeled probe; CaMYB80, 6-FAM labeled probe and 50× unlabeled probe; CaMYB80 and 6-FAM labeled mutant probe. **(d)** LUC analysis results. **(e)** GUS enzyme activity results. Asterisks indicate significant difference (^*^P < 0.05; ^**^P < 0.01).

We first determined the subcellular localization of CaMYB80 and CaPOA1 by co-expressing GFP-CaMYB80 and GFP-CaPOA1 fusion proteins with cytoplasmic and nuclear markers in tobacco leaves. Confocal microscopy observation showed that GFP-CaMYB80 and GFP-CaPOA1 were localized in the nucleus and cytoplasm (Fig. 6a).

To test the hypothesis of direct regulation of *CaPOA1* expression by CaMYB80, we conducted yeast one-hybrid experiments. The results demonstrated normal growth of yeast cells co-expressing prey and bait plasmids on selective media, indicating the ability of CaMYB80 to bind to the MYB binding site in the *CaPOA1* promoter (Fig. 6b). Furthermore, validation through EMSA assay confirmed the binding capability of CaMYB80 to the MYB binding site within the *CaPOA1* promoter (Fig. 6c).

We further verified the Y1H and EMSA results by performing dual luciferase and GUS reporter assays. The results showed that the LUC and GUS activities were significantly higher in tobacco leaves co-infiltrated with 35S:CaMYB80 and CaPOA1pro: LUC/GUS than those infiltrated with 35S: empty and CaPOA1pro: LUC/GUS. The plant luciferase *in vivo* imaging and GUS staining results were consistent with the LUC/GUS activity results (Fig. 6d and e). These results indicate that CaMYB80 specifically binds the *CaPOA1* promoter and activates its transcription.

### *CaPOA1* silencing reduces the cold tolerance of pepper

**Figure 7:**
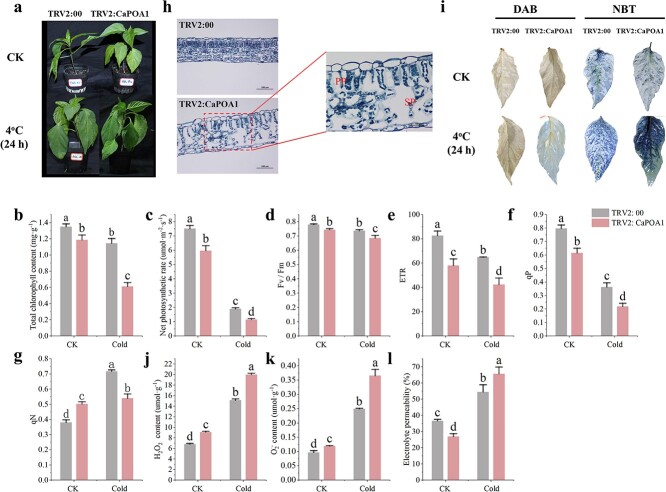
*CaPOA1* silencing reduces the cold tolerance of pepper. Values are means ± SD from three independent experiments. Values with different letters above the bars are significantly different at *P* < 0.05. **(a)** Comparison phenotypes between CaPOA1-silenced plants and control plants. **(b)** Total chlorophyll content. **(c)** Net photosynthetic rate. **(d)** Fv/Fm. **(e)** ETR. **(f)** qP. **(g)** qN. **(h)** Transverse sections of leaves of CaPOA1-silenced plants and control plants at low temperature. PP: palisade tissue; SP: spongy tissue. **(i)** NBT and DAB histochemical staining. **(j)** H_2_O_2_ content. **(k)** O_2_^−^ content. **(l)** Electrolyte permeability.

We validated the cold resistance function of *CaPOA1* using VIGS and conducted RT-qPCR to determine the silencing efficiency of *CaPOA1*, which was found to be close to 76.9% (Fig. S8a, see online supplementary material). Under normal temperature conditions, there was no significant difference in the phenotype of TRV2:CaPOA1 and TRV2:00 plants. However, after 24 h of treatment at 4°C, the leaves of TRV2:CaPOA1 plants showed more severe wilting (Fig. 7a), as demonstrated by a significant decrease in the total chlorophyll, Pn, Fv/Fm, ETR, and qP contents, and a significant increase in qN (Fig. 7b–g). Further leaf anatomy study revealed that TRV2:CaPOA1 plants exhibited a looser cell structure, sparse palisade tissue, and larger gaps between sponge tissue after three days of cold treatment (Fig. 7h).

The histological staining results showed that compared to TRV2:00 plants, TRV2:CaPOA1 plants had more spots on the leaves under normal and low-temperature conditions (Fig. 7i). We quantitatively analysed the levels of H_2_O_2_ and O_2_^−^ (Fig. 7j and k), and the results were consistent with the histological staining results. Moreover, the electrolyte permeability of TRV2:CaPOA1 plants was significantly lower than control plants under normal temperature but significantly higher under low-temperature conditions (Fig. 7i).

Additionally, we conducted measurements of SOD, POD, and CAT activities, along with Pro, Sp, and MDA levels in pepper leaves. The findings revealed markedly decreased levels of SOD, POD, Sp, and Pro, accompanied by a notable increase in MDA level in CaPOA1-silenced plants compared to the control plants. However, there was no significant disparity in CAT activity between the two groups (Fig. S8b–g, see online supplementary material). These outcomes suggest that silencing of *CaPOA1* diminishes the cold tolerance of pepper plants, rendering them more vulnerable to oxidative harm.

### Ectopic expression of *CaPOA1* enhances Arabidopsis cold tolerance

To further investigate the function of *CaPOA1*, we produced transgenic Arabidopsis plants overexpressing *CaPOA1* in the T3 generation. PCR and RT-qPCR were employed to select three transgenic lines (OE6, OE8, and OE10) exhibiting high *CaPOA1* expression levels for freezing tolerance assessment (Fig. S9a and b, see online supplementary material). Remarkably, compared to WT plants, these transgenic Arabidopsis lines displayed notably enhanced survival rates under both NA and CA conditions (Fig. S9c, see online supplementary material).

**Figure 8:**
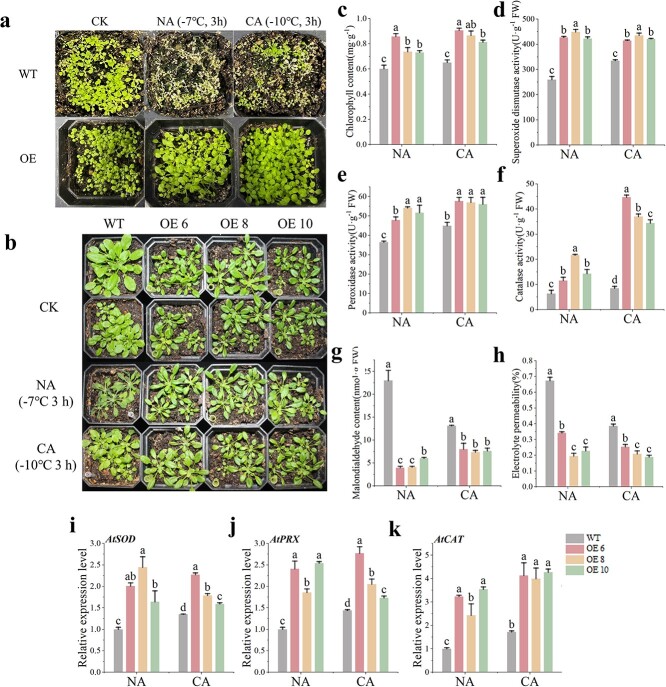
Ectopic expression of *CaPOA1* enhances Arabidopsis cold tolerance. Values are means ± SD from three independent experiments. Values with different letters above the bars are significantly different at *P* < 0.05. **(a)** Survival rate test phenotypes of WT Arabidopsis and transgenic lines (OE6, OE8, OE10). **(b)** Comparison phenotypes between WT Arabidopsis and transgenic lines (OE6, OE8, OE10). **(c)** Total chlorophyll content. **(d)** Superoxide dismutase activity. **(e)** Peroxidase activity. **(f)** Catalase activity. **(g)** Electrolyte permeability. **(h)** Malondialdehyde content. **(i)**–**(k)** Expression levels of *AtSOD*, *AtPRX*, and *AtCAT* in WT Arabidopsis and transgenic lines (OE6, OE8, OE10).

Overexpression of *CaPOA1* significantly enhanced the freezing tolerance (Fig. 8a and b) and increased total chlorophyll content and the SOD, POD, and CAT activities (Fig. 8c–f) of Arabidopsis under NA and CA conditions plants. Further analysis of the MDA content and electrolyte permeability indicated that the membrane damage was less severe in the transgenic Arabidopsis plants overexpressing *CaPOA1* than in the WT plants under cold stress (Fig. 8g and h). We also found that the expression levels of *AtSOD*, *AtPRX*, and *AtCAT* genes in Arabidopsis under NA and CA treatments were consistent with the physiological indexes (Fig. 8i–k). These results suggest that CaPOA1 improves Arabidopsis’ ability to withstand freezing temperatures by enhancing the activities of antioxidant enzymes and the expression of related genes, thereby positively regulating the plant’s response to low-temperature stress.

### *CaMYB80* promotes the expression of the CBF regulatory network-related genes

**Figure 9:**
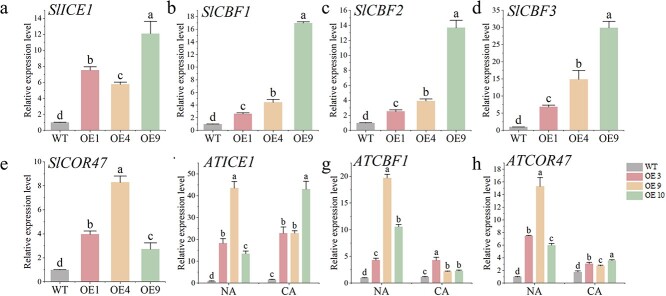
*CaMYB80* promotes the expression of the CBF regulatory network-related genes. **(a)**–**(e)** Expression levels of *SlICE1*, *SlCBF1*, *SlCBF2*, *SlCBF3*, and *SlCOR47* in WT tomatoes and transgenic tomatoes (OE1, OE4, OE9) at low temperature. **(f)**–**(h)** Expression levels of *AtICE1*, *AtCBF1*, and *AtCOR47* in WT Arabidopsis and transgenic lines (OE3, OE9, OE10). Values are means ± SD from three independent experiments. Values with different letters above the bars are significantly different at *P* < 0.05.

To explore the potential regulatory role of *CaMYB80* on genes within the CBF regulatory network, we assessed the expression of *ICE*, *CBF*, and *COR47* genes in both WT and CaMYB80-overexpressing tomato and Arabidopsis plants under NA and CA conditions using RT-qPCR. Notably, the expression levels of cold-responsive genes *SlICE1*, *SlCBF1*, *SlCBF2*, *SlCBF3*, and *SlCOR47* were markedly elevated in CaMYB80-OE tomato plants (Fig. 9a–e). Similarly, in CaMYB80-OE Arabidopsis lines, the expression levels of *ATICE1*, *ATCBF1*, and *ATCOR47* were significantly augmented under both NA and CA treatments (Fig. 9f–h). These findings suggest that CaMYB80 modulates the expression of genes within the CBF regulatory network in response to low-temperature stress, consequently enhancing the plants’ cold tolerance.

## Discussion

As a warmth-loving vegetable, pepper experiences reduced yield and quality and even plant death under low-temperature stress [22]. Therefore, exploring the cold tolerance mechanism of pepper is of great significance for improving its quality and yield. In our investigation, we identified an R2R3-type MYB TF, CaMYB80. Phylogenetic analysis demonstrated that CaMYB80 shares significant homology with other plant R2R3 MYB proteins, particularly showing close relatedness to tomato SlMYB80 (Fig. 1a). Previous research has linked the *Sl*MYB80 gene with cold tolerance in tomato plants [23]. In this article, we reported that CaMYB80 is a cold-responsive TF that enhances plant cold tolerance. We discovered that CaMYB80 interacts with the promoter of Ca*POA1*, increasing Ca*POA1* transcript levels and thereby positively regulating cold tolerance in peppers. These findings provide important insights for the molecular breeding of cold-resistant pepper varieties.

Previous studies have shown that MYB TFs play a crucial role in regulating plant cold resistance [1, 11]. These proteins can regulate plant cold tolerance by regulating the ROS scavenging system. Overexpression of the R2R3-MYB gene *LeAN2* could increase antioxidant enzyme activity and enhance the low-temperature stress tolerance of tomato [24]. Additionally, overexpressing the MYB-related grape TF, *AQUILO*, in Arabidopsis could increase SOD and POD activity and upregulate the expression of their encoding genes [25]. In this experiment, CaMYB80-silenced plants showed a more sensitive phenotype to low temperature (Fig. 2a), while CaMYB80-overexpressing (OE) Arabidopsis and tomato plants showed increased tolerance to low temperature (Figs 3b  and 4a). Damaged Photosystem II (PSII) and reduced chlorophyll content are common in stress-sensitive plants, low-temperature stress can reduce the ability of PSII to utilize light energy, leading to photoinhibition, while also causing significant damage to membrane structures [26]. Under cold stress, the Pn and chlorophyll contents of CaMYB80-silenced plants were significantly lower than those of the control plants (Fig. 2b and c). Simultaneously, there is more damage to leaf structure, resulting in disordered arrangement of cellular tissues (Fig. 2h). However, the chlorophyll content of CaMYB80-OE plants was higher (Fig. 3c). ROS are the products of plant metabolism and have extremely high activity and toxicity [26]. The ROS accumulation was significantly reduced in CaMYB80-OE tomato plants (Fig. 4f) but significantly increased in CaMYB80-silenced plants compared to the control plants (Fig. 2k and l). This finding was confirmed by tissue staining, as CaMYB80-silenced plants had deeper DAB and NBT staining under low-temperature stress (Fig. 2i). In plants, MDA content and electrolyte permeability are indicators of ROS-mediated cell membrane damage [27, 28]. In *chrysanthemum*, the MDA content and relative electrolyte permeability in the *Dg*MYB2-OE lines were the lowest, followed by the WT and *Dg*MYB2-RNAi lines [1]. In our study, the electrolyte permeability and MDA levels of CaMYB80-OE plants were significantly reduced after low-temperature stress compared to the WT plants (Fig. 3g and h). These findings indicate that during cold stress, CaMYB80 can maintain leaf photosynthetic capacity, elevate antioxidant enzyme activity for ROS scavenging, uphold the equilibrium of ROS within cells, and thereby enhance plant cold resistance. These results provide evidence for the positive role of CaMYB80 in response to low-temperature stress.

**Figure 10:**
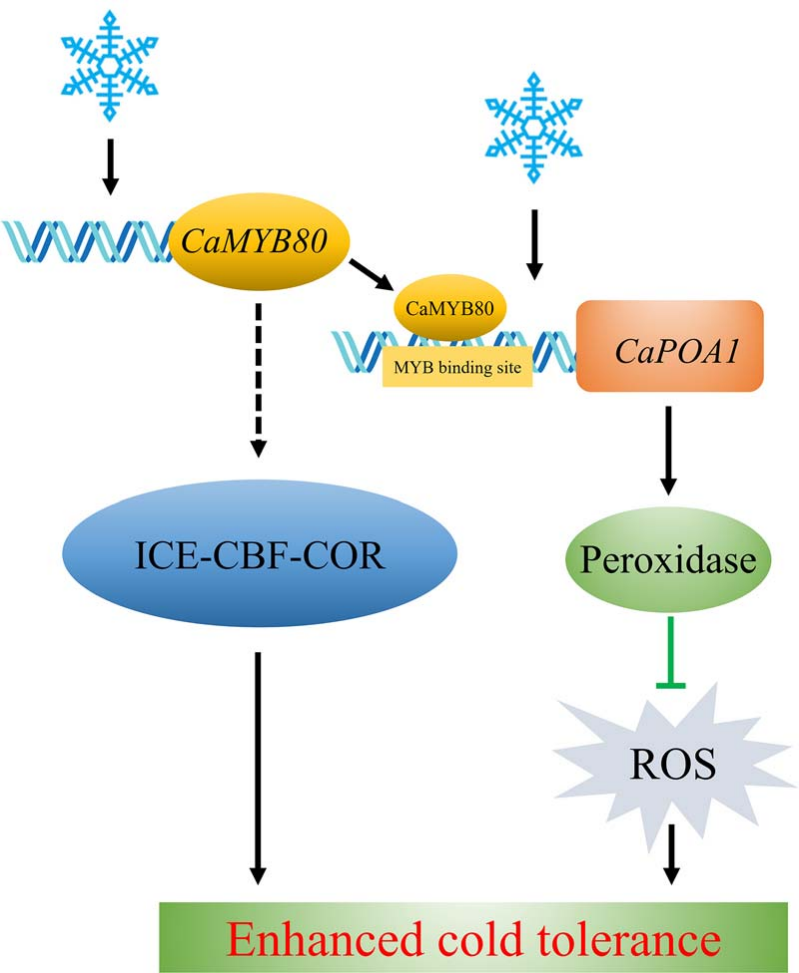
Proposed model through which CaMYB80 enhanced the cold tolerance of pepper by directly targeting *CaPOA1*.

Studies have revealed that MYB TFs predominantly regulate gene expression by binding to the promoter regions of target genes, thus either facilitating or inhibiting their expression [10, 29]. In chrysanthemums, the downstream target gene *DgGPX1* of *Dg*MYB2 has been identified, and overexpression of *DgMYB2* can enhance plant cold tolerance by increasing the activity of glutathione peroxidase. [1]. Similarly, *Md*MYB23 has been found to directly bind to the promoter regions of *MdCBF1* and *MdCBF2*, thereby enhancing apple cold resistance by activating the expression levels of downstream *COR* genes [30]. In pears, *Pbr*MYB5 directly activates the expression of *PbrDHAR2*, consequently enhancing cold tolerance by promoting the synthesis of ascorbic acid (AsA) [31] (Fig. 1g). Our research indicates that the C-terminal of CaMYB80 possesses transcriptional activation activity (Fig. 1g). This attribute may be linked to the characteristic features of MYB transcription factors, where the DNA-binding domain located at the N-terminus distinguishes various types of MYB TFs, while the C-terminus is responsible for mediating transcriptional activity [32]. Through analyses involving Y1H, LUC/REN, GUS activity, and EMSA, our investigation revealed that CaMYB80 exhibits direct binding to the promoter region of the *CaPOA1* gene, and positively regulates the expression of *CaPOA1* (Fig. 6b–e). Additional evidence suggests that POD, an antioxidant enzyme widely present in plants, works synergistically with SOD and CAT to remove excess free radicals under stress conditions, thereby enhancing plant stress resilience [33]. In Arabidopsis, elevating the expression of *At*Prx3 resulted in heightened tolerance to dehydration and salt stress, whereas suppressing *At*Prx3 led to the manifestation of dehydration and salt-sensitive traits [34]. Introducing *CaPOD2* from pepper into Arabidopsis enhances the plant’s resistance to pathogens [35]. Moreover, heterologous expression of *OsPrx114* in rice also enhanced resistance to pathogens by inducing pathogen-related genes [36]. Our results indicated that *CaPOA1* plays a crucial role in clearing excess ROS. Under low-temperature conditions, transgenic Arabidopsis plants overexpressing *CaPOA1* had lower ROS levels, while *CaPOA1*-silenced plants had higher levels of ROS compared to WT plants. Due to the beneficial role of *CaPOA1* under low temperatures, we hypothesize that *Ca*MYB80 can confer cold tolerance. The primary mechanism by which *CaMYB80* enhances plant cold resistance is by enhancing the expression of *CaPOA1* associated with scavenging ROS. In this study, SOD, POD, and CAT activities were lower in CaMYB80-silenced and CaPOA1-silenced plants compared to control plants (Figs S2c–e  and S8b–d, see online supplementary material), indicating that *CaMYB80* and *CaPOA1* silencing reduces ROS levels. Conversely, plants overexpressing *CaMYB80* and *CaPOA1* exhibited higher SOD, POD, and CAT activities than control plants (Figs 3d–f  and 8d–f; Fig. S5a–c, see online supplementary material). Furthermore, the expression of antioxidant-related genes (*AtSOD*, *AtPRX*, and *AtCAT*) significantly increased in CaMYB80-OE plants and CaPOA1-OE plants (Figs 3i–k  and 8i–k). These results support the notion that *CaMYB80* enhances plant cold tolerance by increasing the ability of *CaPOA1* to scavenge ROS molecules (Fig. 10).

Furthermore, previous research indicated that stress-related genes regulate stress responses in transgenic plants [37]. To date, the most well-studied low-temperature regulation network is the ICE-CBF-COR (inducer of CBF expression, C-repeat binding factor, cold-responsive) transcriptional model [38]. Under low temperatures, the dehydration-responsive element-binding 1 (DREB1/CBFs) genes are rapidly induced, followed by activation of downstream target *COR* genes, resulting in the accumulation of protective substances that promote cold acclimation and freezing tolerance, thereby enhancing their cold tolerance [39, 40]. ICE1 can bind the MYC binding site in the *CBF1–3* promoter, thereby promoting *CBF* expression under cold stress and positively regulating plant response to low temperatures [41]. In *AtICE1* mutant plants, *AtCBF* expression is suppressed under cold stress, resulting in reduced cold tolerance in Arabidopsis [42]. In this study, overexpression of *CaMYB80* significantly increased the expression levels of *ICE1*, *CBFs*, and *COR47* in tomatoes and Arabidopsis (Fig. 9a–h). These results suggest that *CaMYB80* may enhance plant cold tolerance by regulating the expression of CBF network genes (Fig. 10).

## Conclusion

Our study presents the inaugural framework elucidating the role of *CaMYB80* in modulating cold tolerance in pepper. The research unveils that (i) *CaMYB80* is directly activated by low temperatures and enhances pepper cold tolerance by upregulating the expression of cold stress-responsive genes (ICE-CBF-COR); (ii) CaMYB80 interacts directly with *CaPOA1*, thereby decreasing ROS accumulation through the promotion of *CaPOA1* expression; and (iii) *CaPOA1* positively regulates cold tolerance in peppers by enhancing ROS scavenging capacity to reduce leaf oxidative damage, thereby maintaining plant tolerance to low-temperature stress. These findings indicate that *CaMYB80* potentially enhances pepper cold tolerance by impacting the CBF pathway and bolstering ROS scavenging capacity.

## Materials and methods

### Plant materials and growth conditions

The experimental materials, including Arabidopsis ecotype Columbia-0, wild-type MicroTOM tomato, tobacco (*Nicotiana benthamiana*), and pepper variety ‘Ganzi’, were obtained from the Vegetable Germplasm Innovation Laboratory of Sichuan Agricultural University. These plants were cultivated in a climate-controlled growth chamber set to a temperature of 25°C during the day and 20°C at night, with a photoperiod of 16 hours of light followed by 8 hours of darkness and maintained at 70% relative humidity.

### Phylogenetic analyses

Target protein sequences (MYB80 and POA1) were obtained through BLAST (Basic Local Alignment Search Tool) on the NCBI website (https://www.ncbi.nlm.nih.gov/). The Neighbor-Joining (NJ) method was used to construct the phylogenetic trees of MYB80 and POA1 in MEGA X software.

### Virus-induced gene silencing of *CaMYB80* and *CaPOA1*

To silence *CaMYB80* and *CaPOA1*, we inserted a 300 bp ORF of each gene into the TRV2 vector, resulting in the generation of recombinant vectors TRV2-CaMYB80 and TRV2-CaPOA1. These vectors, along with TRV2: 00, TRV2: CaPDS, and TRV1 vectors, were transformed into *Agrobacterium tumefaciens* strain GV3101. Subsequently, the TRV2: CaMYB80, TRV2: CaPOA1, TRV2: CaPDS, and TRV2: 00 vectors were mixed equally and infiltrated into the cotyledons of 2-week-old pepper plants. Following two days of incubation in darkness, the plants were transferred to normal growth conditions, following the protocol described by Zhang *et al.* [3]. After 21 days, the efficiency of *CaMYB80* and *CaPOA1* silencing was assessed using RT-qPCR.

### Subcellular localization

The coding sequences (CDS) of the *CaMYB80* and *CaPOA1* genes were amplified using primers designed to exclude stop codons. High-fidelity polymerase chain reaction (PCR) was employed, utilizing Takara high-fidelity enzyme (Takara, Shiga, Japan) for amplification, and the resulting gene sequences were cloned into the PBI221-GFP vector. The GFP-CaMYB80 and GFP-CaPOA1 plasmids, along with the PBI221-GFP empty vector, were individually introduced into *A. tumefaciens* strain GV3101. Bacterial cell suspensions containing different plasmids were mixed at equal ratios of 1:1:1 and infiltrated into tobacco leaves (OD600 = 0.6). The infiltrated plants were maintained in a growth chamber for 2–3 days before being examined and photographed under a laser confocal microscope (FV3000, Olympus, Tokyo, Japan).

### Arabidopsis and tomato transformation

To establish Arabidopsis plants with *CaMYB80* and *CaPOA1* overexpression, we inserted the CDS of *CaMYB80* and *CaPOA1* into the PBI121 vector under the control of the 35S promoter. These constructs, named 35S:CaMYB80 and 35S:CaPOA1, were then introduced into *A. tumefaciens* strain GV3101. Arabidopsis genetic transformation was achieved using the floral dip method, and the resulting T3 plants were selected for subsequent investigations.

For tomato, two-week-old plants underwent transformation via Agrobacterium-mediated leaf infiltration. The infiltrated leaves were utilized to obtain callus tissues, which were cultured on Murashige and Skoog (MS) solid medium supplemented with 50 mg/L kanamycin to encourage elongation and rooting. This process yielded the T0 generation of transgenic tomato plants, which underwent screening to obtain seeds for the T1 generation plants. The transgenic status of both Arabidopsis and tomato plants was confirmed through PCR and RT-qPCR analyses.

### Cold stress tolerance assay

To analyse the cold stress tolerance of the T3 transgenic Arabidopsis and wild-type (WT) Arabidopsis, we performed low-temperature treatment experiments on the WT and transgenic plants expressing *CaMYB80* and *CaPOA1.* The treatments were as follows: (i) CK: normal temperature conditions; (ii) NA: −7°C (3 h) + 4°C (24 h); (iii) CA: 4°C (3 d) + −10°C (3 h) + 4°C (24 h). Following the low-temperature treatment, certain plants were relocated to normal temperature conditions for a 3-day recovery period, and their survival rate was determined. Additionally, to assess the cold tolerance of pepper and tomato plants, experiments involving low-temperature treatments were conducted on pepper plants with silenced CaMYB80 and CaPOA1 genes, CaMYB80-transgenic tomato plants, and WT plants. The treatments were: (i) CK: normal temperature conditions; (ii) 4°C (24 h). After low-temperature stress, samples were taken and stored at −80°C after treatment with liquid nitrogen for later use. Each treatment was set up for three biological replicates.

### Measurement of physiological indicators

The determination details of chlorophyll content, electrolyte permeability, SOD, POD, CAT, MDA, hydrogen peroxide (H_2_O_2_), superoxide anion (O_2_^−^), and Pro were referenced from previous studies [43].Chlorophyll fluorescence parameters were recorded with a PAM2500 chlorophyll fluorometer (Walz, Nuremberg, Germany), and photosynthetic parameters were measured using a LI-6400 portable photosynthesis system (LI-COR Inc., Lincoln, NE, USA).

Plant leaf staining for visualization of H_2_O_2_ and O_2_^−^ contents was performed using diaminobenzidine (DAB) and nitroblue tetrazolium (NBT), respectively. Leaves were immersed in DAB solution (1 mg/ml DAB +20 mM Na_2_HPO_4_, pH 3.8) and NBT solution (0.5 mg/ml NBT), then kept in darkness for 6 hours. Subsequently, photographs were taken (DM2500, Leica, Wetzlar, Germany) after alcohol decolorization.

### RNA extraction and real-time quantitative PCR

Total RNA was extracted from pepper, Arabidopsis, and tomato leaves using the Plant RNA Extraction Kit (Takara, Shiga, Japan). Subsequently, first-strand cDNA synthesis was performed utilizing the PrimeScript kit (Takara, Shiga, Japan), followed by RT-qPCR analysis conducted on an iCycleriQ™ machine (Bio-Rad, Hercules, CA, USA). To determine the relative expression levels of the target genes, normalization was carried out against the geometric mean of Caactin2, Atactin2, and SIUBI3 expression levels using the delta–delta Ct (2^−ΔΔCT^) method. Details of the primers employed for the analysis can be found in Table S1 (see online supplementary material).

### Low-temperature response of *CaMYB80* and *CaPOA1* promoters

To explore the response of *CaMYB80* and *CaPOA1* to low-temperature stress, we constructed CaMYB80pro-LUC, CaMYB80pro-GUS, CaPOA1pro-LUC, and CaPOA1pro-GUS vectors, which were then introduced into *A. tumefaciens* strain GV3101 for co-infiltration into tobacco leaves. The activities of LUC enzyme and GUS enzyme were determined according to Dual-Luciferase Reporter Gene Assay Kit (Beyotime Biotechnology, Shanghai, China) and GUS gene quantitative detection Kit (Coolaber, Beijing, China), respectively.

### *cis*-element analysis

To understand the expression regulation mechanism of *CaSOD*, *CaPOA1*, *CaAPX1*, *CaCAT*, and *CaDHHR2*, sequences upstream of the start codon within a 2000 bp range were extracted, and PlantCARE was used for *cis*-element prediction, with visualization using TBtools.

### Transcriptional activation analysis of CaMYB80

A reporter gene construct was engineered by incorporating 5 × GAL4 sequences and 1 × TATA sequence upstream of the LUC gene within the pGreenII0800-LUC vector. Subsequently, the *CaMYB80* ORF was inserted into an enhanced pSuper1300-YFP vector containing gal4bd, resulting in the creation of the recombinant plasmid pBD-CaMYB80. For comparative analysis, pBD-VP16 served as a positive control, while the improved pBD-Empty vector acted as a negative control. Following transformation, the recombinant plasmids were introduced into *A. tumefaciens* strain GV3101. Co-infiltration of the transformed bacterial solution containing 5 × UAS-TATA-LUC with the bacterial suspensions harboring pBD-CaMYB80, pBD-VP16, and pBD-Empty constructs was conducted in tobacco leaves. The LUC/REN ratio was assessed after 2 days of incubation.

The CDS of *CaMYB80*, along with three sequence fragments (1–63 aa, 64–114 aa, 115–318 aa), were individually inserted into the yeast expression vector pGBKT7. These constructs were subsequently introduced into the yeast strain Y2H and plated onto selective media with or without X-α-gal under double dropout (SD/−Ade/-His) conditions. The plates were then inverted and incubated at 30°C for 3 days, after which colonies were examined and photographed.

### Y1H, dual-LUC, GUS activity, and EMSA assays

The CDS of *CaMYB80* was inserted into the pGADT7 vector, while the anticipated element upstream of the *CaPOA1* promoter was linked to the pAbAi vector for yeast one-hybrid (Y1H) screening. The self-activation of pAbAi-CaPOA1pro using adding aureobasidin A (AbA) (Coolaber, Beijing, China) to SD/-Ura dropout media and the appropriate AbA concentration was screened. Subsequently, pGADT7-CaMYB80 was transformed into competent yeast cells prepared with pAbAi-CaPOA1pro on SD/−Leu dropout media containing AbA (300 ng/mL). After incubation at 28°C in the dark for 2–3 days, the growth of each group was assessed to determine whether there was binding between CaPOA1:pro and CaMYB80.

To further explore the interaction between CaMYB80 and the promoter *cis*-elements of Ca*POA1*, we conducted a luciferase reporter gene assay. The 2000 bp promoter region of Ca*POA1* was cloned into the pGreenII0800-LUC vector to create the reporter construct. Ca*MYB80* was then integrated into the pSuper1300-YFP vector via homologous recombination to generate the effector construct. Subsequently, both the reporter and effector constructs were co-infiltrated into tobacco leaves. Following a 2-day incubation period, *in vivo* imaging was conducted for fluorescence detection, and the LUC/REN ratio was subsequently determined.

GUS activity analysis was conducted by cloning the Ca*MYB80* gene into the pSuper1300-YFP vector and transforming the vector into *A. tumefaciens* strain GV3101 as the effector and cloning the 2000 bp promoter region of Ca*POA1* into the pCAMBIA2300-MCS-GUS vector as the reporter. The reporter and effector were co-infiltrated into tobacco leaves, and after 2 days of incubation, GUS staining and GUS enzyme activity assay were performed on the leaves.

Electrophoresis mobility shift assay (EMSA) was performed according to the method by Yang *et al.* [1]. Briefly, the CDS of *CaMYB80* was cloned into the pCold I vector for HIS-tag fusion, and the HIS-CaMYB80 protein was expressed and purified from BL21 (DE3) cells. The obtained protein was subjected to gel EMSA using the EMSA Chemiluminescent Kit (Thermo Fisher Scientific, 20 148, Waltham, MA, USA). A 5′ biotin-labeled promoter region of the *CaPOA1* (AATAATTAGGTAACC) was used as the probe for the analysis.

### Statistical analysis

The data were organized using Excel 2019 software (Microsoft, Redmond, WA, USA), SPSS23.0 (IBM, New York, NY, USA) as used for statistical analysis at *P* < 0.05 with Tukey’s test. The graphs were plotted using Orign 2019b (Electronic Arts Inc, Redwood, CA, USA) and the data was presented as the mean ± standard deviation, Values with different letters above the bars are significantly different at *P* < 0.05.

## Acknowledgements

This work was supported by breeding research in vegetables (2021YFYZ0022).

## Author contributions

J.X.: Writing – review & editing, Writing – original draft, Formal analysis, Data curation. D.W.: Data curation. L.L.: Data curation. M.X.: Data curation. Y.T.: Investigation. Y.-S.L.: Investigation. B.S.: Data curation. Z.H.: Methodology. Y.Z.: Methodology. H.L.: Conceptualization, Funding acquisition, Writing – review & editing.

## Supplementary Material

Web_Material_uhae219

## Data Availability

The data are presented within the paper and supplementary files.
