# Supplementary material for: CaMYB80 enhances the cold tolerance of pepper by directly targeting CaPOA1
Source: Hortic Res. 2024 Aug 6;11(10):uhae219. doi: 10.1093/hr/uhae219 (PMC11469921; doi:10.1093/hr/uhae219)
Supplement: Web_Material_uhae219 [file web_material_uhae219.zip › Supplementary materials.docx]

**Supplementary materials:**
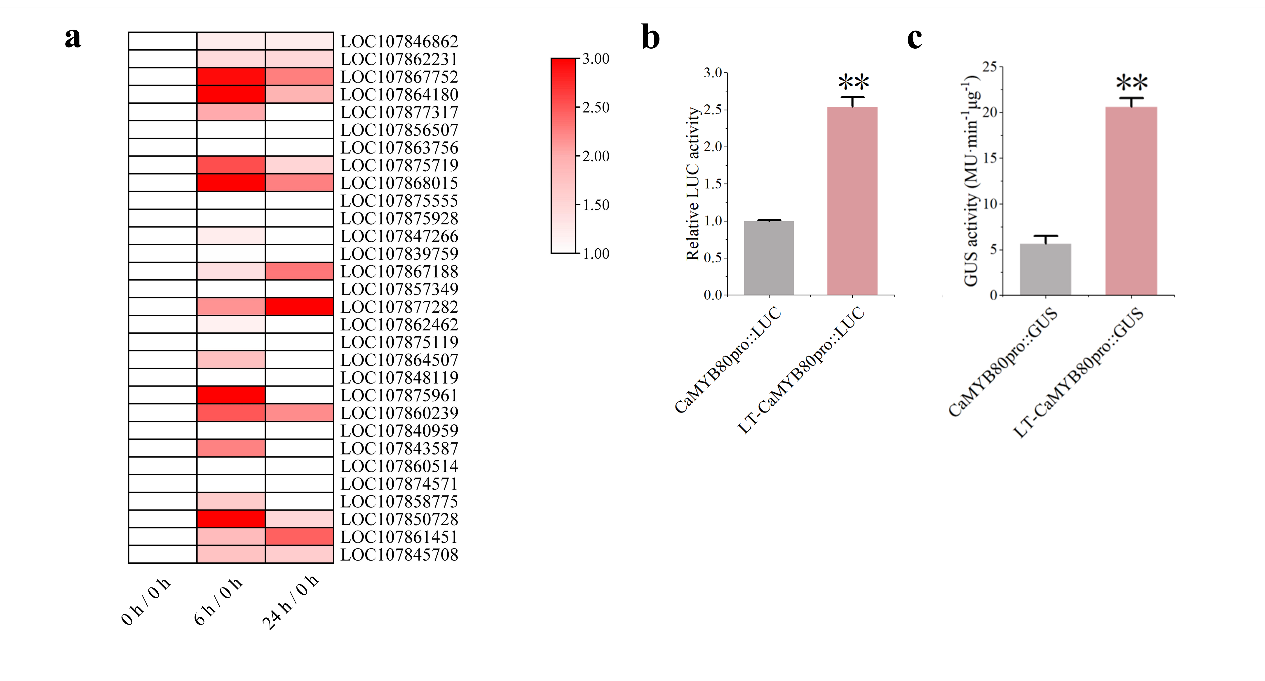


**Figure S1.** *CaMYB80* is responsive to low-temperature stress. Asterisks indicate significant difference (*, *P* < 0.05; **, *P* < 0.01). (a) Transcription levels of 30 MYB transcription factors in pepper at low temperature. (b) Dual-LUC reporter gene assay (DLA) analysis results. (c) GUS enzyme activity analysis results.

**
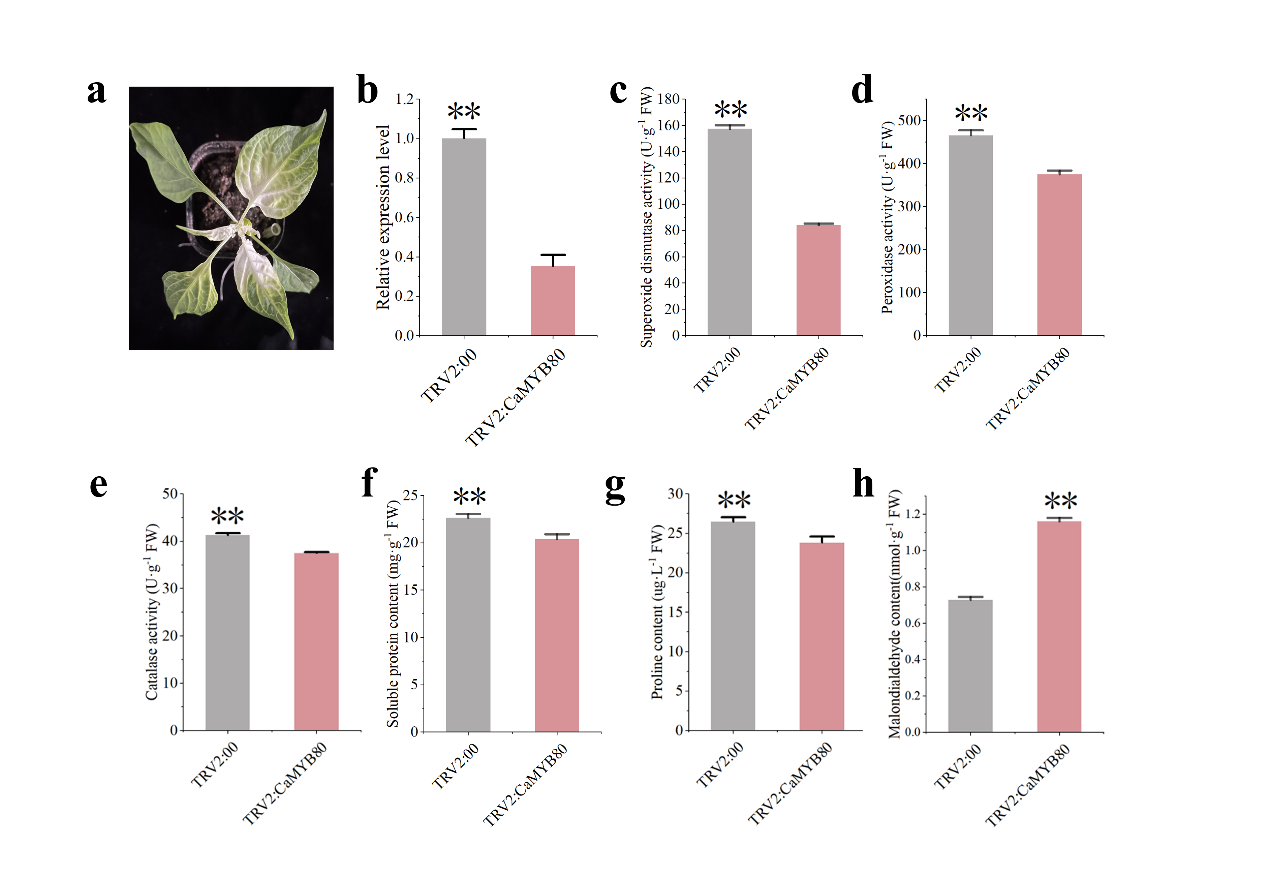
**

**Figure S2.** *CaMYB80* silencing reduces the cold tolerance of pepper. Asterisks indicate significant difference (*, *P* < 0.05; **, *P*< 0.01). (a) The phenotypes of CaPDS-silenced plants. (b)RT-qPCR identification of TRV2-CaMYB80. (c) Superoxide dismutase activity. (d) Peroxidase activity. (e) Catalase activity. (f) Proline content. (g) Soluble protein content. (h) Malondialdehyde content.


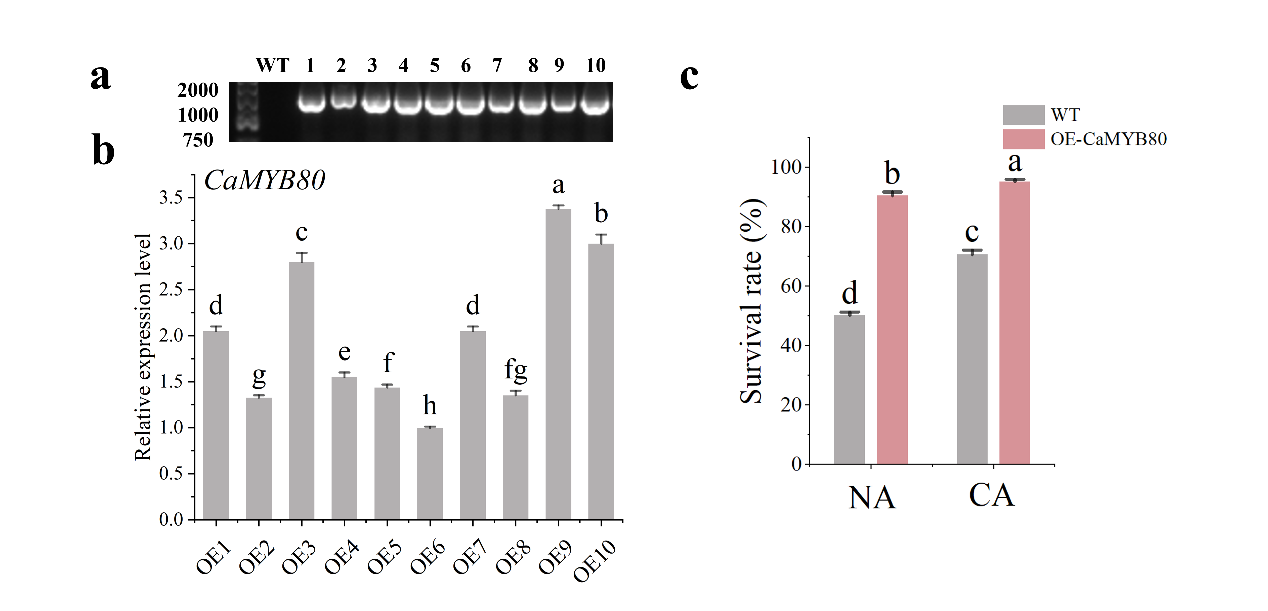


**Figure S3.** (a) PCR identification of transgenic Arabidopsis. (b) RT-qPCR identification of transgenic Arabidopsis. (c) Survival rates of WT Arabidopsis and transgenic lines under NA and CA treatments. Values are means ± SD from three independent experiments. Values with different letters above the bars are significantly different at *P* < 0.05.


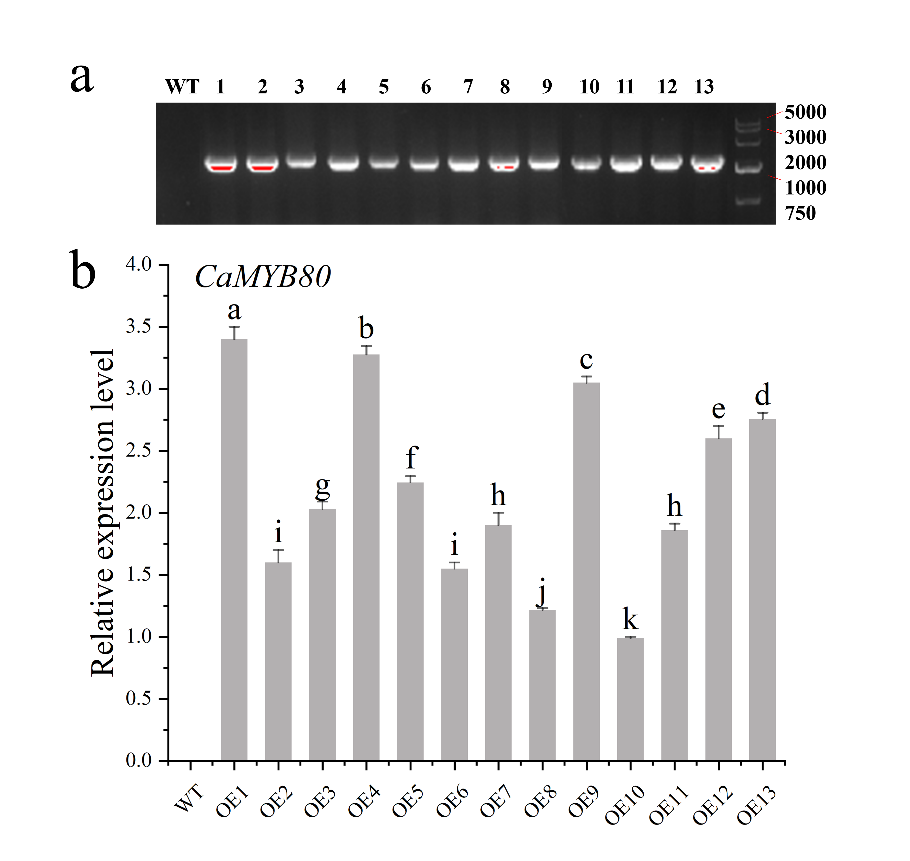


**Figure S4.** (a) PCR identification of transgenic tomato. (b) RT-qPCR identification of transgenic tomato. Values are means ± SD from three independent experiments. Values with different letters above the bars are significantly different at *P*< 0.05.


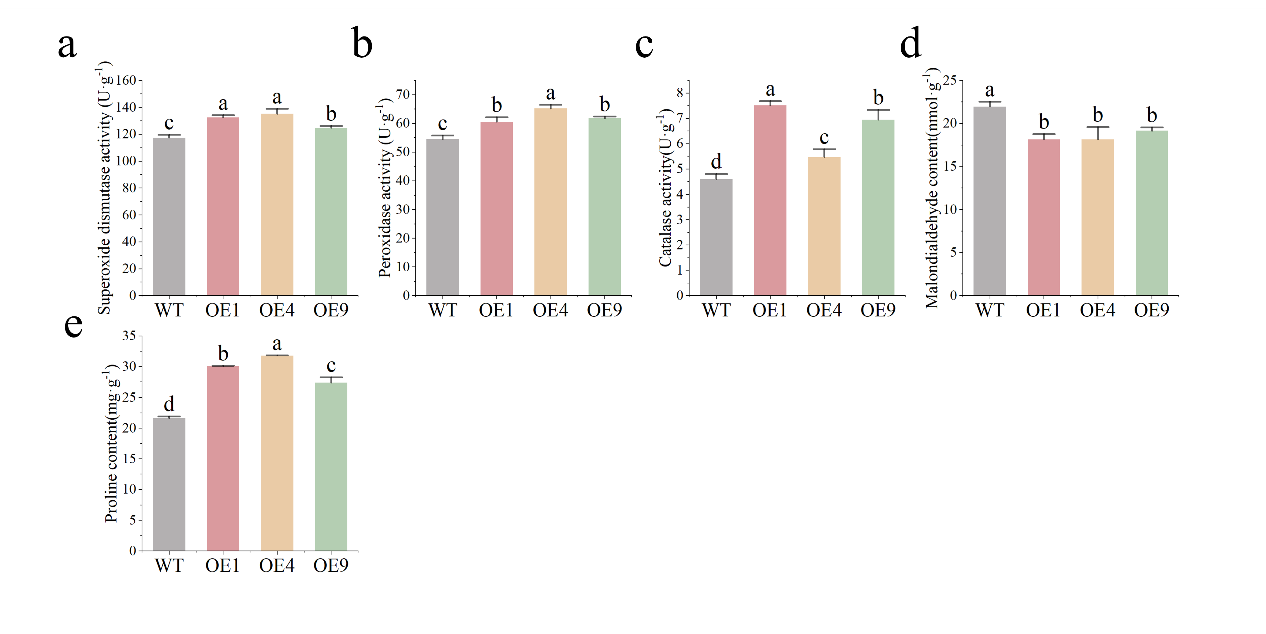


**Figure S5.** Ectopic expression of *CaMYB80* enhances the cold tolerance of tomato. Values are means ± SD from three independent experiments. Values with different letters above the bars are significantly different at *P*< 0.05. (a) Superoxide dismutase activity. (b) Peroxidase activity. (c) Catalase activity. (d) Malondialdehyde content. (e) Proline content.


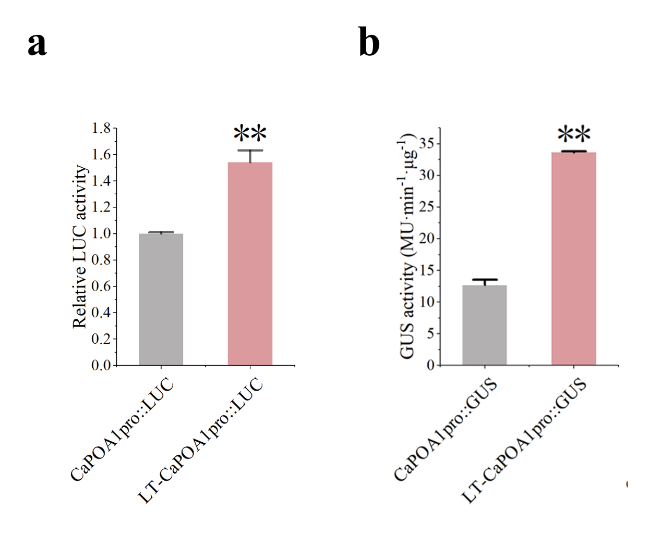


**Figure S6.** *CaPOA1* is responsive to low-temperature stress. Values are means ± SD from three independent experiments. (a) Dual-LUC reporter gene assay (DLA) analysis results. (b) GUS enzyme activity analysis results. Asterisks indicate significant difference (*, *P* < 0.05; **, *P*< 0.01).


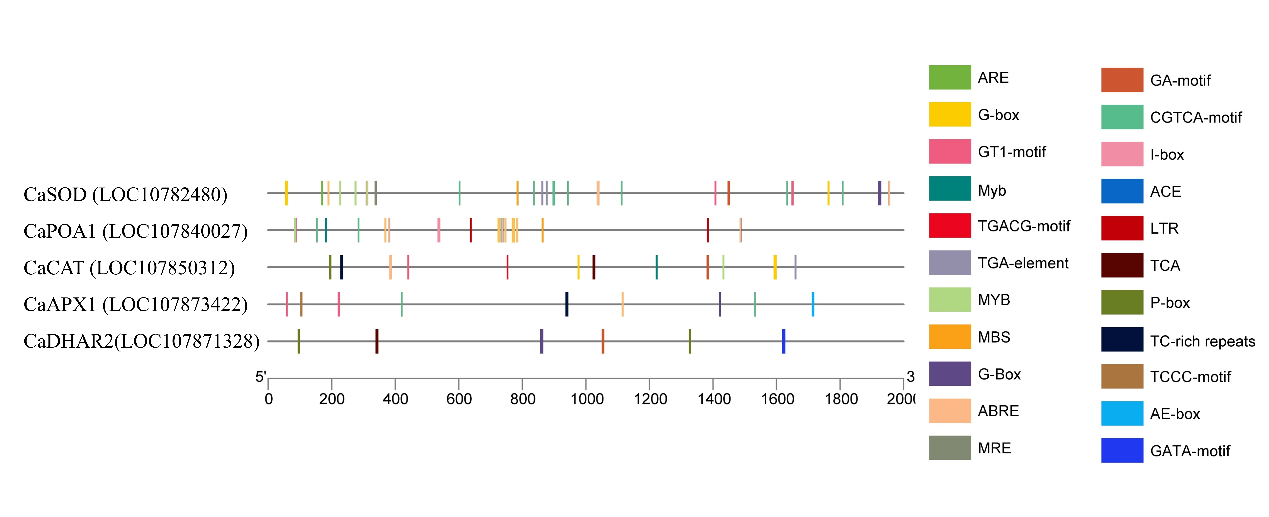


**Figure S7.** Cis-element analysis of promoter regions of *CaSOD*, *CaPOA1*, *CaCAT*, *CaAPX1*, and *CaDHAR2*.


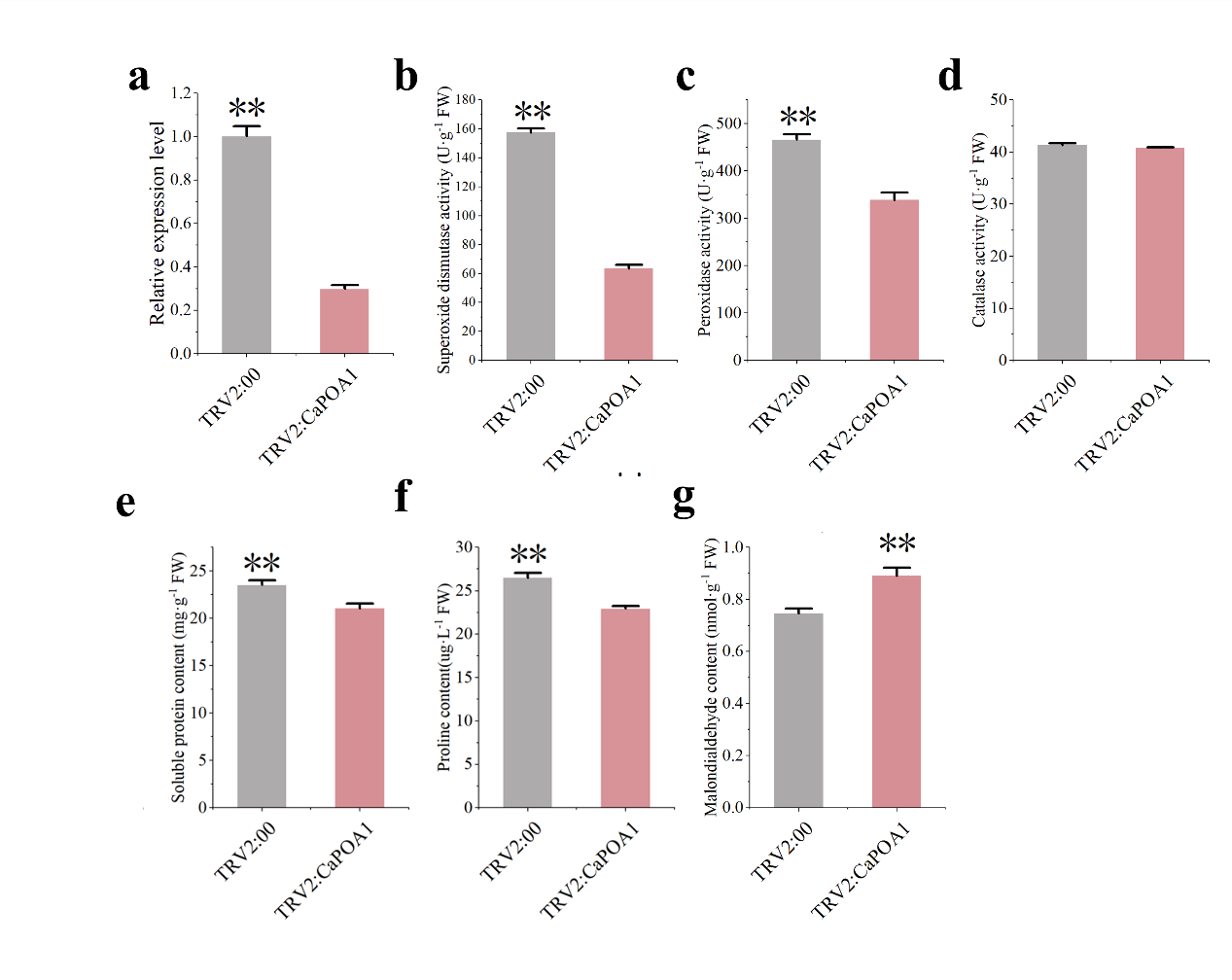


**Figure S8.** *CaPOA1* silencing reduces the cold tolerance of pepper. Asterisks indicate significant difference (*, *P* < 0.05; **, *P*< 0.01). (a) RT-qPCR identification of TRV2-CaPOA1. (b) Superoxide dismutase activity. (c) Peroxidase activity. (d) Catalase activity. (e) Soluble protein content. (f) Proline content. (g) Malondialdehyde content.


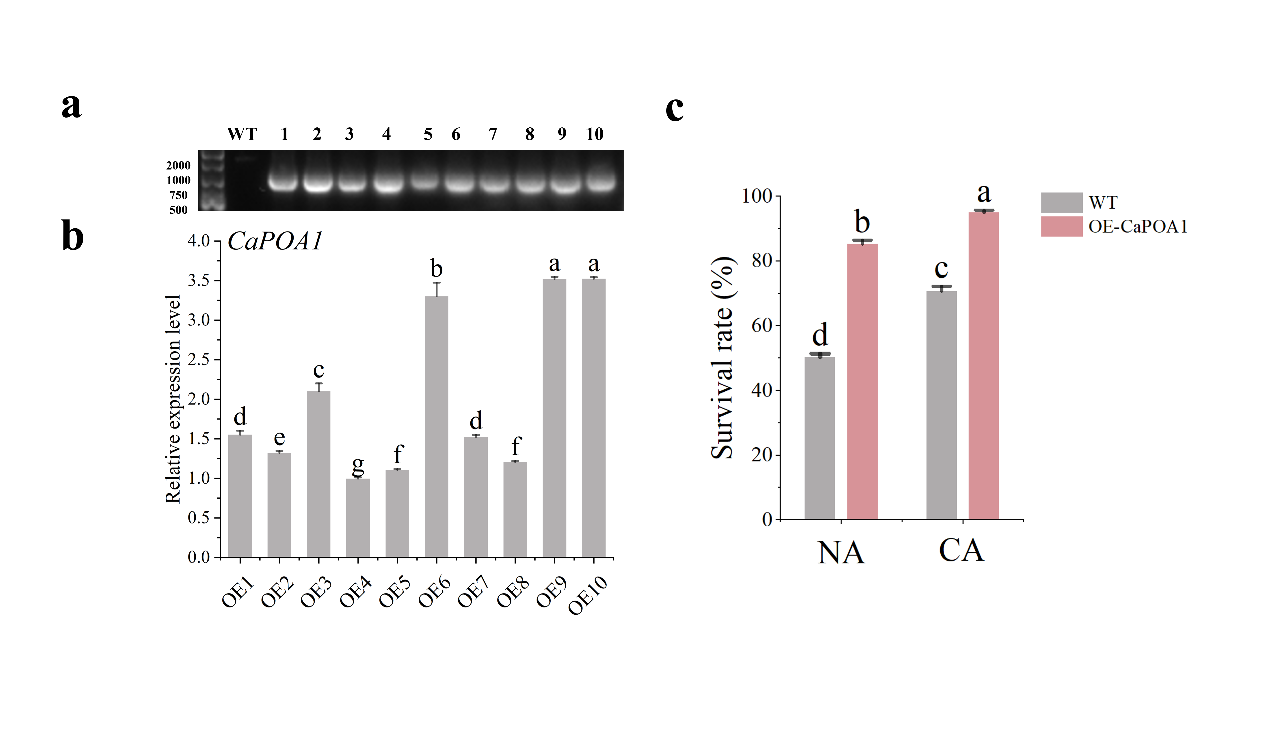


**Figure S9.** (a) PCR identification of transgenic Arabidopsis. (b) RT-qPCR identification of transgenic Arabidopsis. (c) Survival rates of WT Arabidopsis and transgenic lines under NA and CA treatments. Values are means ± SD from three independent experiments. Values with different letters above the bars are significantly different at *P* < 0.05.

**Table S1. Primers were used for the RT-qPCR.**

| **Gene name** | **Gene ID** | **Forward primer** | **Reverse** **primer** |
| --- | --- | --- | --- |
| **CaActin2** | LOC107840006 | TCCACCTCTTCACTCTCTGCTC | TGACCCATCCCTACCATAACAC |
| **CaMYB80** | LOC107845708 | GGGAAGAATTCCATGTTGTGAAAAGGA | TCTGCTTCTGAAAATTGGCCATG |
| **CaPOA1** | LOC107840027 | CAGTACGTGCCCAAGAGCT | GCTGCTTAGCATCATCGATAACC |
| **AtCBF1** | AT4G25490 | GTTTGGGATGCCGACTTTGTTGG | GTCACCATCTCCTTCGCCGTCAT |
| **AtCBF2** | AT4G25470 | CGTTTTATATGGATGAAGAGGC | TAGCTCCATAAGGACACGTCAT |
| **AtCBF3** | AT4G25480 | AGGATGAGATGTGTGATGCG | CCATAACGATACGTCGTCATC |
| **AtCOR47** | AT1G20440 | CAGTGTCGGAGAGTGTGGTG | ACAGCTGGTGAATCCTCTGC |
| **AtSOD** | AT3G11580 | GTTTGGAGCTGGCTGGGC | CTGGCACTTACAGCTTCCCAA |
| **AtPOD** | AT1G67960 | AACACTGTCTCTTGTGCTGACG | GTTGTCGGGTTCAGGAAGGT |
| **AtCAT** | AT1G20620 | GCAACTACCCCGAGTGGAAA | TGTTCAGAACCAAGCGACCA |
| **AtActin2** | AT3G18780 | TAACAGGGAGAAGATGACTCAGATCA | AAGATCAAGACGAAGGATAGCATGAG |
| **AtICE1** | AT3G26744 | CTCTTGTCCTTCTTGGCATTG | TCCTAAAGGCCAGCAAGCTA |
| **SlActin2** | Solyc11g005330 | GTCCTCTTCCAGCCATCCAT | ACCACTGAGCACAATGTTACCG |
| **SlCBF1** | LOC543826 | AATAAGAAGACAAGGATTTGGCT | CGCCTTTTGAATATCTTTAGAGTTGG |
| **SlCBF2** | LOC101263186 | GATTCAGTTTGGAGGTTGCC | GTCACTACTCTCTTGCACATTT |
| **SlCBF3** | LOC109119806 | GGAGTGAGGAAGAGGAATTCT | AATATCCTTAGTGTCAGAGGAATCT |
| **COR47-like** | Solyc04g082200 | AGCAGCTCTAGTAGCTCCAGT | CCTCCGTCTTCTTATGCCCG |
| **SlUBI3** | Solyc01g056940 | TCTTCCGACACCATCGACAA | AGAACTGCAACACAGTGAGC |
| **SlICE1** | LOC101266110 | ACCCAATGGATAACAGCTTC | CCATTAGCAGAATTGTTAGCCC |
